# Determinants of Eligibility and Timing of Autologous Transplantation in Multiple Myeloma: The Role of R-MCI and Diagnostic Plasma Cell Burden

**DOI:** 10.3390/diagnostics15233038

**Published:** 2025-11-28

**Authors:** Ozlem Candan, Arda Bayar, Narmin Naghizada, Derya Demirtas, Ahmet Mert Yanik, Asu Fergun Yilmaz, Ayse Tulin Tuglular, Tayfur Toptas, Isik Atagunduz

**Affiliations:** 1 Division of Hematology, Department of Internal Medicine, Marmara University School of Medicine, Pendik Training and Research Hospital, 34899 Istanbul, Turkey; 2 Department of Internal Medicine, Marmara University School of Medicine, Pendik Training and Research Hospital, 34899 Istanbul, Turkey

**Keywords:** multiple myeloma, autologous stem cell transplantation, transplant timing, comorbidity index, R-MCI, real-world study

## Abstract

**Background:** Autologous stem cell transplantation (ASCT) remains a cornerstone in the management of multiple myeloma (MM). However, the optimal timing of ASCT and the factors determining whether patients ultimately undergo transplantation remain unclear in real-world practice. The Revised Myeloma Comorbidity Index (R-MCI) was developed to quantify patient fitness but its influence on transplant eligibility and timing has not been fully characterized. **Methods:** We conducted a retrospective single-center study including 137 patients with newly diagnosed MM between 2015 and 2025. Clinical parameters recorded at diagnosis included age, sex, performance status, renal and pulmonary function, cytogenetic risk, International Staging System (ISS) stage, and bone marrow plasma cell infiltration. The R-MCI was calculated for all patients. Transplant timing was categorized as early (≤12 months) or delayed (>12 months) after diagnosis. Logistic regression analysis was performed, and variables with *p* < 0.10 in univariate analyses were included in the multivariate model. Early versus delayed ASCT was defined as ≤12 months or >12 months from diagnosis, respectively. **Results:** ASCT was performed in 61/137 patients (44.5%), while 42.6% of these underwent early transplantation. Transplanted patients were significantly younger (<65 years: 82.0% vs. 25.0%, *p* < 0.001) and had lower R-MCI scores (median 0 vs. 1, *p* < 0.001) compared with non-transplanted patients, while plasma cell infiltration and ISS stage did not differ. In multivariate analysis, R-MCI was the only variable showing a trend toward predicting early transplantation (OR 0.27, 95% CI 0.07–1.06, *p* = 0.06). **Conclusions:** In real-world MM management, patients quantified by R-MCI appear to play a more prominent role than disease burden in determining both eligibility for and timing of ASCT. Incorporating comorbidity indices alongside ISS may enhance individualized transplant decision-making and optimize treatment outcomes.

## 1. Introduction

Multiple myeloma (MM) is a plasma cell malignancy accounting for approximately 10% of all hematologic neoplasms. It is characterized by clonal expansion of malignant plasma cells in the bone marrow, leading to end-organ damage such as bone lysis, anemia, renal insufficiency, and immune dysfunction. Over the past two decades, the therapeutic landscape has been transformed by the advent of proteasome inhibitors, immunomodulatory agents, and monoclonal antibodies, markedly improving survival and depth of response. A roadmap towards improving outcomes in multiple myeloma highlights how these novel agents have shifted standard-of-care paradigms [1].

Despite these advances, autologous stem cell transplantation (ASCT) remains a foundational therapy for transplant-eligible patients, offering deeper remission and longer progression-free survival when integrated into modern regimens. The 2024 update on diagnosis, risk-stratification, and treatment emphasizes that even in the era of potent induction therapies, ASCT retains a critical role [2].

The optimal timing of autologous stem cell transplantation (ASCT) in newly diagnosed multiple myeloma (MM) has been debated for decades. Clinical trials have typically endorsed early ASCT—performed within 12 months of diagnosis following induction therapy—as standard practice, citing improvements in response depth and progression-free survival [3]. However, in real-world settings, practice often diverges from trial protocols. Some patients face delays due to comorbidities, organ dysfunction, or suboptimal response to induction therapy [4]. Others confront logistical or socioeconomic hurdles, leading to postponed ASCT timing. In these scenarios, transplantation may be deferred, raising key questions about which clinical or biological factors most significantly contribute to treatment delay [2,5].

Current international guidelines continue to emphasize the importance of early autologous stem cell transplantation (ASCT) in transplant-eligible patients, yet they provide limited direction on how to prioritize or risk-stratify candidates based on the optimal timing of the procedure [6]. To address this gap, several studies have investigated clinical predictors that may influence transplant timing, including disease burden at diagnosis—such as bone marrow plasma cell infiltration—disease stage, cytogenetic abnormalities, renal function, and patient-related characteristics such as age and performance status [7].

The Revised Myeloma Comorbidity Index (R-MCI) was developed to quantify the cumulative comorbidity burden in multiple myeloma (MM) and to support individualized treatment planning. R-MCI incorporates five parameters—age, Eastern Cooperative Oncology Group (ECOG) performance status, renal function (eGFR), pulmonary function, and cytogenetic risk profile—creating a concise, disease-specific score that predicts outcomes more accurately than generic indices [8]. Validated across independent MM cohorts, R-MCI has shown strong predictive value for overall survival and treatment tolerance and is increasingly used to guide therapy intensity in both clinical trials and real-world settings [9]. However, its specific role in defining the timing of transplantation—rather than transplant eligibility alone—remains underexplored.

One particularly understudied factor is the percentage of bone marrow plasma cells at diagnosis, often used as a surrogate for disease burden. While studies have shown that high plasma cell infiltration (e.g., ≥60%) is independently predictive of poorer progression-free survival and overall survival—even after adjusting for established prognostic markers—its direct influence on clinical decisions regarding early versus delayed ASCT remains unproven [10]. Likewise, although transplant-timing studies, such as those by Blackburn et al., provide insights into predictive factors and barriers in real-world settings, the independent and combined roles of comorbidity scores, ISS staging, and plasma cell burden in dictating ASCT timing have not been conclusively delineated [4].

Moreover, it remains uncertain whether the same clinical variables decisively influence the decision to proceed with ASCT at all. In routine clinical practice, a notable proportion of transplant-eligible patients may never receive transplantation due to clinical frailty, comorbidities, or failure to achieve disease control. For example, studies exploring barriers to ASCT report that logistic, financial, and cultural factors can discourage both physicians and patients from pursuing transplant [11]. In addition, data from Tang et al. [12] indicate that among transplant-eligible myeloma patients, those who declined or were deemed unfit for ASCT had worse survival outcomes, emphasizing the clinical importance of understanding who proceeds to transplant and who does not.

In this retrospective observational study, we aimed to evaluate both transplant eligibility and timing in patients with multiple myeloma. Specifically, we assessed whether plasma cell infiltration at diagnosis, R-MCI score, ISS stage, age, and other clinical characteristics are associated with the likelihood of receiving ASCT. Among those who underwent transplantation, we further analyzed which of these variables influenced whether ASCT was performed early (≤12 months) or delayed (>12 months) after diagnosis. By exploring both the eligibility and timing dimensions, this study seeks to clarify the real-world determinants of ASCT decision-making and help inform individualized treatment planning in clinical practice.

## 2. Materials and Methods

This retrospective observational study was conducted at Marmara University Pendik Training and Research Hospital, a tertiary referral center located in Istanbul, Turkey. Adult patients diagnosed with MM between January 2015, and June 2025 were identified through institutional records. Ethical approval was obtained from the local institutional review board, and the study was conducted by the principles of the Declaration of Helsinki.

A total of 150 patients diagnosed with MM between January 2015, and June 2025 were identified. Of these, 13 were excluded due to missing data on one or more key clinical variables (age, ECOG performance status, eGFR, DLCO, cytogenetic profile, or bone marrow plasma cell percentage), yielding a final analysis cohort of 137 patients. Among them, 61 patients underwent ASCT, while 76 did not receive transplantation during follow-up. All patients had received standard induction regimens containing proteasome inhibitors and/or immunomodulatory agents before assessment for transplant eligibility.

For patients who underwent ASCT, the time interval between initial diagnosis and the transplantation procedure was calculated. Transplant timing was categorized as either early (≤12 months from diagnosis) or delayed (>12 months), based on current clinical practice patterns and literature-supported thresholds [13]. This categorization reflects real-world clinical dynamics in which transplant timing may be influenced by disease stabilization, treatment response, or comorbidity burden.

Clinical and laboratory variables recorded at diagnosis comprised age, sex, ECOG, bone marrow plasma cell infiltration percentage, ISS stage, eGFR, and diffusing capacity of carbon monoxide DLCO. Cytogenetic risk status (standard vs. high risk, e.g., del(17p), t(4;14), t(14;16)) was documented when available. These parameters were then used to calculate the R-MCI according to the scoring scheme outlined in Table 1. Total R-MCI scores ranged from 0 to 9 and were stratified as low risk (0–3), intermediate risk (4–6), or high risk (7–9). These scores were then incorporated, alongside the other clinical variables, into subgroup comparisons and multivariate logistic regression analyses to identify predictors of ASCT eligibility and timing.

All statistical analyses were performed using IBM SPSS Statistics (version 26.0; IBM Corp., Armonk, NY, USA). Continuous variables were expressed as medians with interquartile ranges (IQR), and categorical variables as counts with corresponding percentages. Between-group comparisons were conducted using the Mann–Whitney U test for continuous variables and the chi-square or Fisher’s exact test, as appropriate, for categorical variables. Variables with a *p*-value < 0.10 in univariate analyses were entered into the multivariate logistic regression model to identify independent predictors of transplant timing. The linearity assumption for continuous predictors was evaluated by examining functional form and potential transformations, and plasma cell infiltration was retained as a continuous variable, as model fit did not improve with categorization. A two-sided *p*-value < 0.05 was considered statistically significant.

The study protocol was approved by the Marmara University Faculty of Medicine Ethics Committee for Drug and Non-Drug Medical Device Research (Protocol No: 09.2025-25-0796) and conducted in accordance with the Declaration of Helsinki.

## 3. Results

### 3.1. Patient Characteristics

A total of 150 patients diagnosed with multiple myeloma between January 2015 and June 2025 were identified, of whom 137 were included in this retrospective analysis after excluding 13 patients with missing data on key clinical variables. The median age at diagnosis was 64 years (IQR: 57–71), and 57.7% (*n* = 79) were female. The median bone marrow plasma cell infiltration at diagnosis was 60% (IQR: 30–80). Based on the ISS, 26 patients (19.0%) were classified as Stage I, 36 (26.3%) as Stage II, and 64 (46.7%) as Stage III. Staging information was unavailable in 11 patients.

R-MCI, incorporating age, performance status, renal and pulmonary function, and cytogenetic risk, was calculated in all patients. The median R-MCI score was 0 (IQR: 0–1), indicating a predominantly low-risk cohort.

ASCT was performed in 61 patients (44.5%), whereas 76 patients (55.5%) did not undergo transplantation during follow-up. All patients received standard induction therapy, which included proteasome inhibitors and/or immunomodulatory agents, before transplant evaluation.

### 3.2. Comparison Between Transplanted and Non-Transplanted Patients

**Figure 1:**
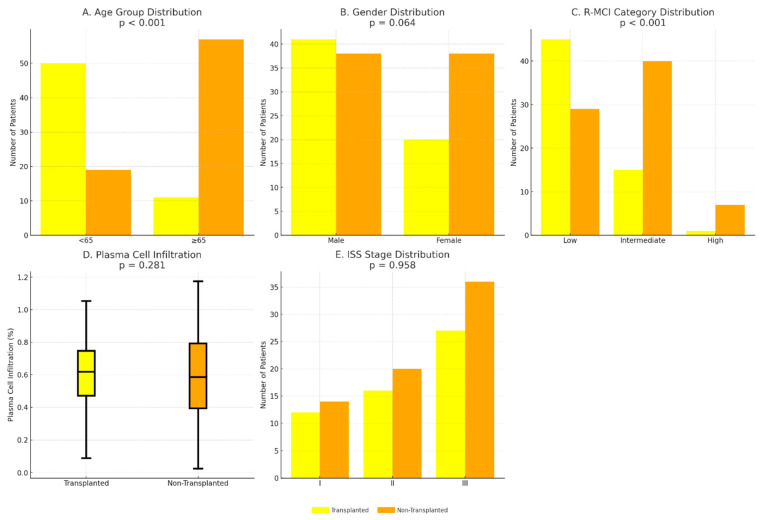
Comparison of clinical and laboratory characteristics between transplanted and non-transplanted patients. (**A**). Age group distribution (<65 vs. ≥65 years; *p* < 0.001). (**B**). Gender distribution (*p* = 0.064). (**C**). Distribution of R-MCI categories (Low, Intermediate, High; *p* < 0.001). (**D**). Plasma cell infiltration (%) in bone marrow aspirates (*p* = 0.281). (**E**). Distribution of ISS stages (*p* = 0.958). *p*-values were calculated using chi-square test for categorical variables and Mann–Whitney U test for continuous variables.

The proportion of patients aged <65 years was significantly higher in the ASCT group than in the non-transplanted group (82.0% vs. 25.0%, *p* < 0.001), indicating that younger age strongly determined transplant eligibility. Similarly, the median R-MCI score was significantly lower among transplanted patients (0 [IQR 0–1] vs. 1 [IQR 0–2], *p* < 0.001), highlighting the influence of comorbidity burden on the decision to proceed with ASCT. In contrast, no significant differences were observed between the two groups in terms of female sex distribution (33% vs. 50%, *p* = 0.064), bone marrow plasma cell infiltration (median 60% vs. 57%, *p* = 0.281), or ISS stage distribution (*p* = 0.958). These comparisons are summarized in Table 2 and illustrated in Figure 1A–E.

### 3.3. Transplant Timing: Early vs. Delayed ASCT

Among the 61 patients who underwent ASCT, 26 (42.6%) received transplantation within 12 months of diagnosis (early ASCT), while 35 (57.4%) had delayed transplantation (more than 12 months).

No univariate differences were observed in age (median 57 vs. 60 years; *p* = 0.677), R-MCI score (median 0 vs. 1; *p* = 0.235), plasma cell infiltration (median 60% vs. 70%; *p* = 0.615), or sex (*p* = 0.989).

**Figure 2:**
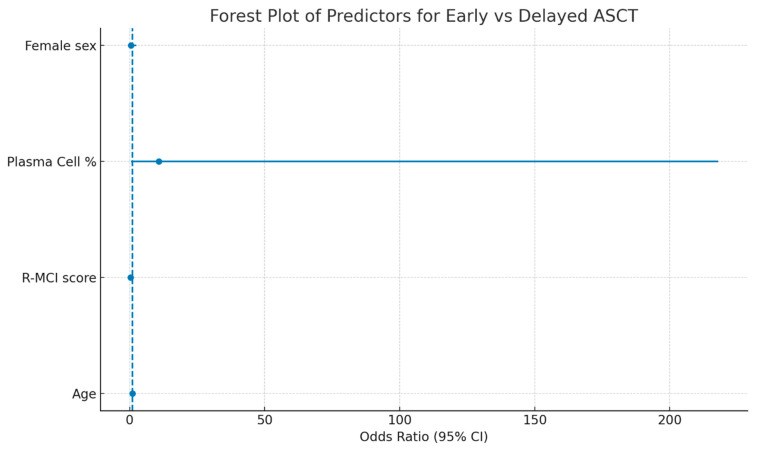
Forest plot of multivariate logistic regression predictors for early versus delayed ASCT. Odds ratios (ORs) and 95% confidence intervals (CIs) are shown for Age, R-MCI score, Plasma Cell percentage, and Female sex. The vertical dashed line indicates OR = 1. Note: The vertical dashed line represents the null value (OR = 1), and solid horizontal lines indicate the 95% confidence intervals for each predictor.

In the multivariate logistic regression analysis (Table 3, Figure 2), the R-MCI score showed a trend towards an association with ASCT timing. Each one-point increase in R-MCI was associated with lower odds of early transplantation; however, this association did not reach statistical significance (OR 0.267; 95% CI 0.067–1.058; *p* = 0.060). Age, plasma cell percentage, and sex were not significantly associated with transplant timing.

## 4. Discussion

In this comprehensive single-center cohort, our data demonstrate that patient-related characteristics—particularly age and comorbidity burden—are the primary drivers of both eligibility for and timing of ASCT. The marked difference in transplant rates between patients aged <65 and ≥65 years (82.0% vs. 25.0%, *p* < 0.001) suggests that chronological age remains a potent, though imperfect, surrogate for physiological reserve in clinical decision-making. More importantly, the significantly lower R-MCI scores among transplanted patients (median 0 vs. 1; *p* < 0.001) indicate that structured comorbidity indices may offer a more nuanced assessment of patient fitness beyond age alone. These findings are consistent with recent literature emphasizing functional and organ-specific assessments over rigid age cut-offs in transplant algorithms [9,14,15].

Contrary to expectations based on historical trial data, neither baseline tumor burden—reflected by bone marrow plasma cell infiltration—nor ISS stage distribution differed significantly between groups, suggesting that modern induction regimens may attenuate the prognostic weight of initial disease load when selecting transplant candidates [16]. The broad confidence interval for plasma cell infiltration likely reflects sample size limitations and the skewed distribution of infiltration values; therefore, this result should be interpreted cautiously. The variable was retained in the model due to its clinical relevance in MM and its potential influence on ASCT decision-making.

Although lower R-MCI scores appeared numerically more common among patients undergoing earlier ASCT, this association was not statistically significant, as the confidence interval included unity and the *p*-value slightly exceeded the significance threshold. The relatively small number of transplanted patients may have further limited the statistical power to detect a meaningful association. In the multivariate analysis, higher R-MCI scores were similarly associated with reduced odds of early transplantation, although this finding again did not reach statistical significance (OR 0.267; 95% CI 0.067–1.058; *p* = 0.060). Taken together, these observations suggest that comorbidity burden may influence not only the decision to proceed with ASCT but also the timing of high-dose therapy, potentially guiding clinicians in optimizing patients before transplantation [17,18,19].

Our findings highlight a potential role for R-MCI in shared decision-making discussions. By quantifying comorbidity burden, clinicians can more transparently communicate risks and benefits to patients, tailor pre-transplant interventions (e.g., rehabilitation, nutritional support), and set realistic expectations regarding timing and outcomes [20,21]. Although our study did not reveal a statistically significant association between ISS stage and the decision to proceed with transplantation, this does not diminish the prognostic importance of ISS; in fact, current international guidelines continue to strongly recommend early ASCT in patients with high ISS stage [2,6].

Our results suggest that in physiologically fit patients with preserved organ function and good performance status, the recommendation for early ASCT in high ISS disease remains highly relevant; however, with modern therapies, this decision can be further individualized by incorporating factors such as comorbidity profile, depth of treatment response, and patient preference [16,22]. The deep remissions and organ function recovery achieved with contemporary induction regimens may partially mitigate the adverse impact of high disease stage on transplant candidacy. Therefore, while ISS remains a critical determinant in transplant planning, integrating it with comorbidity indices such as R-MCI, depth of response, and patient preference could support a more nuanced and patient-centered approach to ASCT timing and eligibility [23,24,25].

Interestingly, baseline bone marrow plasma cell percentage did not differ significantly between transplanted and non-transplanted patients, nor between early and delayed ASCT groups. This finding may reflect the impact of contemporary, highly effective induction regimens—particularly regimens based on proteasome inhibitors (PIs) and immunomodulatory drugs (IMiDs), and more recently, triplet or quadruplet combinations incorporating the anti-CD38 monoclonal antibody daratumumab—which can produce rapid and deep cytoreduction before ASCT evaluation. Induction with bortezomib, lenalidomide, and dexamethasone (VRd) has been shown to achieve high rates of ≥VGPR and substantial reductions in bone marrow plasma cell burden after only 3–4 cycles [26], while daratumumab-based regimens further increase the depth of early response and rates of measurable residual disease (MRD) negativity in transplant-eligible patients [27]. Such rapid tumor debulking may diminish the prognostic weight historically attributed to baseline marrow plasma cell infiltration when determining ASCT eligibility and timing. Therefore, in the modern treatment era, the depth of response and recovery of organ function achieved with induction therapy may be at least as influential as initial tumor burden in shaping real-world transplant decisions [26,27].

Taken together, these observations highlight the complementary value of integrating structured comorbidity assessment tools such as the R-MCI alongside established disease staging systems like the ISS to individualize ASCT planning. Such an approach may ensure that transplant candidacy and timing reflect not only disease burden but also each patient’s unique physiological reserve and treatment response. Future prospective multicenter studies incorporating robust survival endpoints and comprehensive molecular risk stratification will be instrumental in validating these findings and informing next-generation transplant timing algorithms.

### Limitations

This study’s retrospective, single-center design introduces potential selection bias and limits generalizability across diverse practice settings. Cytogenetic and molecular risk data were not consistently available, potentially underrepresenting the impact of high-risk abnormalities on transplant decisions. This study lacked detailed information on induction regimens, depth of treatment response, and measurable residual disease status, all of which are recognized contributors to ASCT eligibility and timing. The absence of these parameters may have influenced the associations observed in our analysis. Additionally, we did not evaluate long-term outcomes—such as progression-free or overall survival—relative to the timing of ASCT, which restricts interpretation of the clinical benefit associated with early versus delayed transplantation. Socioeconomic factors, depth of response to induction therapy, logistical constraints, and individual patient preferences were not captured in our dataset. They may have importantly influenced both the eligibility and scheduling of ASCT. Despite these limitations, our findings provide valuable real-world insights into the pivotal role of comorbidity assessment in transplant decision-making.

Key strengths of this study include its real-world cohort, systematic assessment of both ASCT eligibility and timing, and the incorporation of the R-MCI score into transplantation decision analysis. Future studies with larger sample sizes, prospective designs, and integration of induction regimen details, depth of response, and MRD evaluation will be essential to validate and refine these observations.

## 5. Conclusions

In real-world practice, patient fitness—defined by age and comorbidity burden via the R-MCI—appears to play a more prominent role than traditional disease burden metrics in determining both eligibility for and timing of ASCT in multiple myeloma. Although our study did not demonstrate a statistically significant association between ISS stage and transplant decisions, this finding does not diminish the prognostic significance of ISS; current guidelines continue to strongly recommend early ASCT in patients with high ISS stage. Our results suggest that in physiologically fit patients with preserved organ function and good performance status, the recommendation for early ASCT in high-ISS disease remains highly relevant; however, with modern therapies, this decision can be further individualized by incorporating factors such as comorbidity profile, depth of treatment response, and patient preference.

Incorporating structured comorbidity assessment tools into transplant decision workflows, alongside established disease staging systems, may enhance individualized patient selection and optimize the timing of high-dose therapy. Prospective, multicenter studies incorporating comprehensive molecular profiling and survival analyses will be instrumental in developing new transplant decision-making algorithms that integrate both R-MCI and ISS.

## Figures and Tables

**Table 1:** Calculation of the Revised Myeloma Comorbidity Index (R-MCI).

Parameter	Condition	Score
Age	≤50 years	0
	51–60 years	1
	61–70 years	2
	>70 years	3
ECOG performance status	0–1	0
	2	1
	≥3	2
Renal function (eGFR)	≥60 mL/min/1.73 m^2^	0
	30–59 mL/min/1.73 m^2^	1
	<30 mL/min/1.73 m^2^	2
Pulmonary function (DLCO)	≥65% predicted	0
	50–64% predicted	1
	<50% predicted	2
Cytogenetic risk profile	Standard risk	0
	High-risk abnormalities *	1

ECOG: Eastern Cooperative Oncology Group; eGFR: estimated glomerular filtration rate; DLCO: diffusing capacity of carbon monoxide. * High-risk cytogenetics include del(17p), t(4;14), t(14;16), etc. Risk categories: Low risk (0–3); Intermediate risk (4–6); High risk (7–9).

**Table 2:** Comparison of Patient Characteristics Between Transplanted and Non-Transplanted Patients.

Variable	Transplanted (*n* = 61)	Non-Transplanted (*n* = 76)	*p*-Value
Age <65, *n* (%)	50 (82.0%)	19 (25.0%)	<0.001
Female, *n* (%)	20 (33%)	38 (50%)	0.064
R-MCI, median (IQR)	0 (0–1)	1 (0–2)	<0.001
Plasma Cell %, median (IQR)	60 (36–80)	57 (20–80)	0.281
ISS Stage I, *n* (%)	12 (19.7%)	14 (18.4%)	
ISS Stage II, *n* (%)	16 (26.2%)	20 (26.3%)	
ISS Stage III, *n* (%)	27 (44.3%)	36 (47.4%)	0.958

IQR = interquartile range; R-MCI = Revised Myeloma Comorbidity Index; ISS = International Staging System; *n* = number of patients. *p*-values are calculated using Mann–Whitney U test for continuous variables and chi-square test for categorical variables.

**Table 3:** Logistic Regression Results for Predictors of Early ASCT (≤12 Months).

Variable	Univariate OR	Univariate 95% CI	Univariate *p*-Value	Multivariate OR	Multivariate 95% CI	Multivariate *p*-Value
Age	0.987	0.915–1.064	0.726	1.028	0.939–1.126	0.549
R-MCI score	0.383	0.124–1.184	0.096	0.267	0.067–1.058	0.060
Bone marrow plasma cell (%)	1.549	0.170–14.129	0.698	10.797	0.535–217.883	0.121
Female sex	0.635	0.189–2.130	0.462	0.539	0.120–2.427	0.421

ASCT = autologous stem cell transplantation; OR = odds ratio; CI = confidence interval; R-MCI = Revised Myeloma Comorbidity Index. *p*-values are calculated using Mann–Whitney U test for continuous variables and chi-square test for categorical variables.

## Data Availability

The datasets generated and analyzed during the current study are available from the corresponding author upon reasonable request. Due to patient privacy and ethical restrictions, data is not publicly available.
